# Comprehensive application of bio-char and nitrogen fertilizer in dry-land maize cultivation

**DOI:** 10.1038/s41598-022-16971-0

**Published:** 2022-08-05

**Authors:** Chen Sun, Jiying Sun, Julin Gao, Jian Liu, Xiaofang Yu, Zhigang Wang, Xiujuan Yang, Nan Ji

**Affiliations:** College of Agronomy, Inner Mongolia Agricultural University, No. 275, XinJian EastStreet, Hohhot, 010019 China

**Keywords:** Physiology, Plant sciences

## Abstract

Drought stress and the scarcity of nitrogen fertilizer are two of the important abiotic factors affecting maize growth. Bio-char can enhance the maize yield. Therefore, two field experiments were carried out in the 2 years (2019–2020) to study the effects of nitrogen fertilizer at three levels and four levels of bio-char on endogenous protective enzymes, dry matter accumulation, and yield of the maize ‘Xianyu 335’ under two different irrigation methods. A split-plot system in three replications was established to conduct the field trials. Two irrigation methods (Regular irrigation and Irregular irrigation) were in the main plots, three nitrogen fertilization levels (0, 150, 300 kg h^−1^ m^2^) were in sub-plots, and four bio-char levels (0, 8, 16, 24 t h^−1^ m^−2^) were in the sub-sub plots. Each sub-plot consisted of 9 rows with 5 m length and 0.6 m width, and each sub-plot area was 30 m^2^ in the 2 years. The results indicated that the irrigation methods, the nitrogen, and bio-char supply significantly affected the maize endogenous protective enzymes, dry matter accumulation, and yield in the 2 years. Under the same irrigation method, nitrogen fertilizer and bio-char significantly improved the endogenous protective enzyme activity, dry matter accumulation, and yield of maize compared to the treatment without nitrogen fertilizer and bio-char. The above characteristics improved with increased bio-char supply and nitrogen fertilization at 150 kg h^−1^ m^−2^. The treatment of C24N150 recorded the highest values for the parameters of maize endogenous protective enzymes activity, dry matter accumulation, and yield under different irrigation methods during the two harvest seasons.

## Introduction

As one of the staple crops, maize has a high food, feeding, and industrial value^1^. Maize yield formation is regulated not only by gene expression and photosynthesis but also by a combination of exogenous cultivation measures and environmental factors^2^. Inner Mongolia is located north of the 200 mm equivalent rainfall line and belongs to the arid-semi-arid zone^3^. Drought is one of the leading environmental stressors limiting dry matter accumulation and yield formation in maize^4^. Bio-char, as an exogenous substance with special structure and stable properties, can effectively improve water, fertilizer, air, and heat conditions in the cultivated layer, create soil climate cycle, and enhance endogenous protective enzyme activity and maize yield^5–8^. Since bio-char itself is physically and chemically stable and not suitable for self-decomposition or degradation by microorganisms, it can exist in the tillage layer for a long time and continue to be effective^9,10^. As a major element, nitrogen contributes significantly to dry matter accumulation, yield formation, and regulation of endogenous protective enzymes in maize as well^11,12^. Different amounts of nitrogen application cause different effects of regulation^13^. Excessive nitrogen application will not only cause yield reduction but also reduce nitrogen utilization efficiency and produce the nitrogen enrich ment phenomenon^14–16^. According to Wang^17^, moderate nitrogen fertilization can enhance superoxide dismutase and peroxidase activity so that damage to cells caused by reactive oxygen radical ions can be mitigated. In addition, the yield at relatively low N levels was significantly higher than that at relatively high N levels^18^. Meanwhile, Fu^19,20^ also suggested that medium N application had the most significant effect on the regulation of dry matter accumulation and yield formation in maize.

In summary, previous studies have mainly focused on the effects of bio-char on soil amendment effectiveness and yield as well as the effects of nitrogen on maize growth, development, and yield formation^21–23^. However, there was a lack of research on the cooperation of different irrigation methods, nitrogen fertilization levels, and bio-char levels. Accordingly, on the basis of the previous studies^24–27^, 2-year field experiments were conducted to determine the effects of bio-char and nitrogen supply on endogenous protective enzymes, dry matter accumulation, and yield of maize under different irrigation conditions in the Tumochuan Plain area in Midwestern Inner Mongolia of China. The main objective of this study was to investigate how different nitrogen fertilization levels and bio-char levels could influence maize growth and yield under different irrigation conditions. To be specific, we tested (1) how different bio-char levels and nitrogen fertilization levels influenced endogenous protective enzymes, dry matter accumulation, and yield of maize under regular irrigation and irregular irrigation conditions? (2) What was the best treatment for achieving high yield? (3) What was the best method for maximizing the yield of maize without irrigation conditions? (4) Whether the effects of the treatments varied over the 2 years? The information obtained in this study will contribute to the refinement of high-yielding cultivation models as well as provide a theoretical approach for tackling drought stress.

## Materials and methods

### Site description

Two field experiments were carried out at the experimental base of Inner Mongolia Agricultural University (40° 33′ N, 110° 31′ E) located in the Midwestern Inner Mongolia of China during the seasons from 2019 to 2020. The 2-year experiments were carried out in the same plot, the test of nitrogen fertilizer was started in 2019, while the bio-char was applied as a one-off before the seeding stage in 2017. The surface soil fertility (0–20 cm) and the climatic conditions during the growth period (from April 1 to October 31) of maize were shown in Table 1. The maize was seeded on April 27 and 24 and was harvested on October 7 and 10 in 2019 and 2020, respectively. Base fertilizer applied at the seeding included P_2_O_5_ and K_2_O at the rate of 105 kg h^−1^ m^−2^ and 35 kg h^−1^ m^−2^, respectively. In addition, the urea of different application levels of 0, 150, and 300 kg h^−1^ m^−2^ were top-dressed in different experimental treatment cells during the V12 in 2019 and 2020. A series of cultivation and management measures such as weeding and pest control were carried out according to the local high-yield cultivation^28^.

**Table 1:** Soil fertility and climatic conditions.

Year	Available N (mg kg^−1^)	Available P (mg kg^−1^)	Available K (mg kg^−1^)	Organic matter (g kg^−1^)	pH	Sunshine hour (h)	Average temperature (°C)	Average rainfall (mm)	Monthly average rainfall (mm)
2019	48.64	2.3	112.02	15.59	8.2	1893.6	22.1	213.5	52.29
2020	48.25	2.19	112.61	15.69	8.2	1825.7	21.8	235.7	48.06

### Experimental design

A split-plot design with three replications was used. The two irrigation methods (regular irrigation and irregular irrigation) were assigned in the main plots, which were expressed by RI and II. The three nitrogen fertilization levels (0, 150, and 300 kg h^−1^ m^−2^) were allocated in the sub-plots, which were expressed by N0, N150, and N300. The four bio-char levels (0 t h^−1^ m^−2^, 8 t h^−1^ m^−2^, 16 t h^−1^ m^−2^, and 24 t h^−1^ m^−2^) were appointed in sub-sub plots, which were expressed by C0, C8, C16, and C24. The treatments of this experiment were as follows: C0N0, C8N0, C16N0, C24N0, C0N150, C8N150, C16N150, C24N150, C0N300, C8N300, C16CN300, and C24N300.

The test material was maize ‘Xianyu 335’, which was half horse-toothed and sold in Chinese markets. The heavy-duty type precision seeder (2BMJ-9, Heilongjiang, KVERNELAND Agricultural Machinery Co., Ltd., Heilongjiang, China) was used to seed. The planting density was 82,500 plants h^−1^ m^−2^, and each sub-plot consisted of 9 rows with 5 m length and 0.6 m width, and each sub-plot area was 30 m^2^ in the 2 years.

The plots of regular irrigation were irrigated four times during the growth period (V6, V12, R1, and R2), and the plots of irregular irrigation were irrigated only once at V6. Specifically, the irrigation date of V6, V12, R1 and R2 was June 24, July 8, July 22, August 3, and June 26, July 10, July 24, and August 6 in 2019 and 2020, respectively. The irrigation interval was 14 days. The irrigation volume was 750 m^3^ at a time, and it was controlled by an Intelligent electromagnetic flow converter (ZEF, Liaoning, Dalian Zhongyang Auto-control Technology Co., Ltd. Liaoning, China).

### Measurements

Malondialdehyde content (MDA)^29^: a total of 0.5 g of maize leaves along with 5 ml of 5% TCA was ground into homogenate and then centrifuged (3000 r, 10 min) with a high-speed centrifuge (Z383K, Shanghai, HERMLE Instrument Technology Co., Ltd., Shanghai, China). A total of 2 ml of supernatant liquid and 2 ml of 0.67% TBA were taken and added into a centrifuge tube, then boiled in a water bath at 100 °C for 30 min, then centrifuged after cooling to room temperature. The absorbance values of the supernatant at 450 nm, 532 nm, and 600 nm were determined, respectively.

$$\text{MDA \, concentration } (\upmu \text{mol}/\text{L})=6.45\cdot (\text{A}_{532}-\text{A}_{600})-0.56\cdot \text{A}_{450}$$

In the equation above, A_450_, A_532_, and A_600_ represented absorbance values at wavelengths of 450 nm, 532 nm, and 600 nm, respectively.

$$\text{MDA content }(\upmu \text{mol}/\text{g FW})=\text{C}\cdot \text{V}/\text{W}$$

In the equation above, V and W were represented as the extracted liquid volume and the fresh weight of the sample, respectively.

Superoxide dismutase activity (SOD)^30,31^: a total of 0.5 g of maize leaves along with 4 ml of pre-cooled phosphate buffer was grounded into homogenate, Then, 2 ml of homogenate was taken into a centrifuge tube and centrifuged (4000 r,10 min), so that supernatant liquid obtained was enzyme liquid. Four 5 ml finger tubes were taken, two of them were test tubes and the others were control tubes. A total of 1.5 ml of 0.05 mol l^−1^ phosphate buffer, 0.3 ml of 130 mmol l^−1^ Met solution, 750 μmol l^−1^ NBT solution, 0.3 ml of 100 μmol l^−1^ EDTA-Na2 solution, 0.3 ml of 20 μmol l^−1^ riboflavin solution, 0.05 ml of enzyme liquid and 0.25 ml of distilled water was added into test tubes, respectively.

A total of 1.55 ml of 0.05 mol l^−1^ phosphate buffer, 0.3 ml of 130 mmol l^−1^ Met solution, 750 μmol l^−1^ NBT solution, 0.3 ml of 100 μmol l^−1^ EDTA-Na2 solution, 0.3 ml of 20 μmol l^−1^ riboflavin solution and 0.25 ml of distilled water was added into control tubes, respectively. One of the control tubes was selected and placed in a dark place while the other tubes reacted under 4000 Lx sunlight for 20 min. At the end of the reaction, the tube in darkness was used as a reference to determine the absorbance values of the other tubes.

$${\text{Superoxide dismutaseactivity }}\left( {{\text{U}}/{\text{g}}} \right) = \left( {{\text{A}}_{{{\text{CK}}}} - {\text{A}}_{{\text{E}}} } \right) \cdot {\text{V}}/\left( {0.{5} \cdot {\text{A}}_{{{\text{CK}}}} \cdot {\text{W}} \cdot {\text{V}}_{{\text{t}}} } \right)$$

In the equation above, A_CK_, A_E_, V, V_t_, and W represented the absorbance values of the light control tube, absorbance values of the test tubes, the total volume of the sample solution, sample consumption during the measurement, and the fresh sample quality, respectively.

Peroxidase activity (POD)^32^: a total of 5 g of maize leaves along with the proper amount of phosphate buffer was grounded into homogenate. Then, the homogenate was taken into a centrifuge tube and centrifuged (3000 r, 10 min). The supernatant liquid obtained was added to a 25 ml volumetric flask. The remaining sediment was prepared as a suspension with 5 ml phosphate buffer, and then centrifuged (3000 r, 10 min) two times, the supernatant liquid obtained was incorporated into a volumetric flask and diluted with buffer solution to volume. A total of 2.9 ml of 0.05 mol l^−1^ phosphate buffer along with 1 ml of 2% H_2_O_2_ and 1 ml of 0.05 mol l^−1^ guaiacol was added into two centrifuge tubes, respectively. 0.1 ml of enzyme liquid was transferred into a centrifuge tube while inactivated enzyme liquid (boiled in a water bath for 5 min) was transferred into another centrifuge tube as a control. Incubated at 37 °C water bath for 15 min, then transferred to an ice bath and 2 ml of 20% trichloroacetic acid was taken to terminate the reaction immediately. The absorbance was measured at 470 nm with a visible-light spectrophotometer (SP-723, Shanghai Spectrum Instruments Co., Ltd., Shanghai, China) after centrifugation (3000 r, 10 min).

$${\text{Peroxidaseactivity}}\left[ {{\text{U}}/\left( {{\text{g}}\;{\text{min}}} \right)} \right] = \Delta {\text{A}}_{{{47}0}} \cdot{\text{V}}_{{\text{T}}} /\left( {{\text{W}}\cdot{\text{V}}_{{\text{S}}} \cdot0.0{1}\;{\text{t}}} \right)$$

In the equation above, △A_470_ is the change of absorbance during the reaction period, V_T_ is the total volume of the extracted enzyme liquid, W is the fresh weight of the sample, V_S_ is the enzyme liquid volume taken for determination, and t is the reaction time.

Dry matter accumulation^33^: maize plants were taken in each plot during the R1 and R6 stages with three replicates. Maize plants were dried at 105 °C for 30 min, then dried at 80 °C to constant weight, and weighed the dry matter weight.

Ylild^34^: at the physiological maturity stage (R6), two rows in the middle of the measured production area were selected, and all plants in two rows were harvested after the removal of the side plants, ten plants with uniform ear growth were selected for determination of row grains, ear rows, 100-grain weight, and grain water content (measured by an LDS-1G moisture content detector), then calculated the 1000-grain weight and maize yield.

### Statistics analysis

Microsoft Excel 2016 (Microsoft, Inc., Redmond WA, USA) and the SAS 9.4 statistical software (SAS Institute Inc., CA, USA,) were used to finish the statistical analysis. The correlation analysis was carried out using Data SPSS window version 22 (SPSS Inc., Chicago, USA). The Sigmaplot 14 (Systat Software Inc., San Jose, CA, USA,) was used to create figures.

## Results

### Significance tests of irrigation methods, nitrogen fertilization levels, bio-char dosage and their interactions

Analysis of variance (ANOVA) results showed that irrigation methods, nitrogen fertilization, and bio-char levels significantly affected maize endogenous protective enzymes, dry matter, and yield from 2019 to 2020 (Table 2). Significant interactions between irrigation methods and nitrogen fertilization levels were found in superoxide dismutase activity, peroxidase activity, malondialdehyde content and dry matter in R1 of 2019, and malondialdehyde content, dry matter in R1 and yield of 2020. Significant interactions between irrigation methods and bio-char levels were found on superoxide dismutase activity, peroxidase activity, malondialdehyde content, dry matter in R1 and yield of 2019, and superoxide dismutase activity, peroxidase activity, malondialdehyde content and dry matter in R1 of 2020. Significant interactions between nitrogen fertilization levels and bio-char levels were only found on malondialdehyde content and dry matter in R1 of 2019. Through the comparison of 2-year F-values, it could be found that the effect of irrigation methods on maize endogenous protective enzymes, dry matter, and yield was greater than that of nitrogen fertilization levels and bio-char levels.

**Table 2:** Significance of the effects of irrigation methods, nitrogen fertilization levels, bio-char levels and their interactions on maize growth and yield using ANOVA.

Year	Source	SOD activity (U mg^-1^)	POD activity (μ g^−1^ min^−1^)	MDA content (μmol g^−1^)	Dry matter in R1 (g plant^−1^)	Dry matter in R6 (g plant^−1^)	Yield (kg h^−1^ m^−2^)
2019	I	290,164.16**	70,886.58**	9.60**	70,699.28**	208,532.59**	10,097,100.5**
	N	6107.70**	5892.70**	0.73**	20,169.25**	36,629.26**	10,393,276.3**
	B	4855.22**	1208.81**	0.44**	7946.72**	9950.65**	7,380,317.5**
	I*N	948.53**	320.23**	0.64**	1895.76**	3.07 ns	45,308.0 ns
	I*B	1486.48**	133.95*	0.04**	467.99**	190.79 ns	348,143.1*
	N*B	323.26 ns	46.77 ns	0.02**	297.16*	192.56 ns	154,434.3 ns
	I*N*B	256.61 ns	62.31 ns	0.03**	32.77 ns	272.11 ns	53,392.3 ns
2020	I	397,884.12**	69,708.33**	7.03**	87,983.62**	228,038.68**	131,641,538.0**
	N	14,928.20**	5084.57**	0.90**	29,624.76**	41,972.59**	11,665,380.5**
	B	6027.17**	1090.46**	0.34**	7807.38**	9053.22**	7,129,577.7**
	I*N	785.32 ns	47.20 ns	0.30**	901.68**	351.13 ns	1,137,105.8**
	I*B	737.31*	181.47**	0.16**	731.83**	178.31 ns	221,938.0 ns
	N*B	81.50 ns	47.19 ns	0.01 ns	70.41 ns	198.04 ns	29,515.3 ns
	I*N*B	89.10 ns	25.03 ns	0.01 ns	26.93 ns	180.49 ns	26,164.0 ns

Numbers were F values. Stars indicated the level of significance (*p < 0.05, **p < 0.01), ns represented insignificant. I represented irrigation methods, including RI and DS; N represented nitrogen fertilization levels, including N0, N150,N300; B represented bio-char levels, including C0,C8,C16,C24.

### Effects of bio-char and nitrogen fertilizer on malondialdehyde content of maize under different irrigation methods

**Figure 1:**
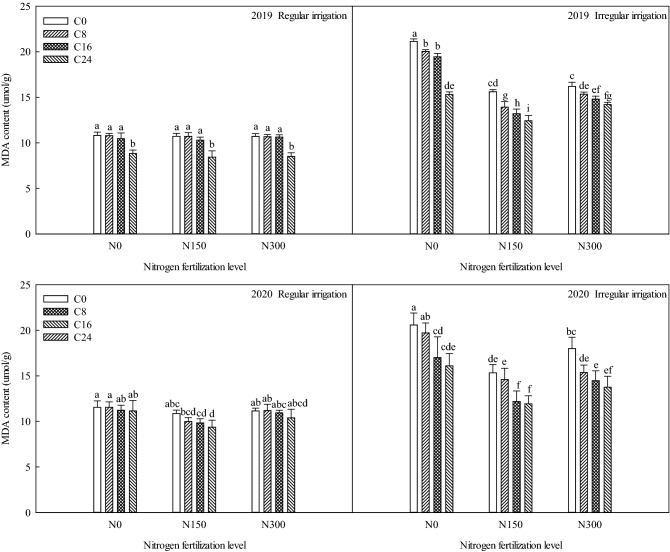
Effects of bio-char and nitrogen fertilizer on MDA content of maize under different irrigation methods.

Figure 1 shows that under the regular irrigation condition, the malondialdehyde content stayed at a steady level. Under the irregular irrigation condition, the nitrogen fertilization levels and bio-char levels significantly influenced (p ≤ 0.05) the malondialdehyde content compared to C0N0, resulting in the malondialdehyde content values that were lower than that of C0N0. Nitrogen and bio-char supply decrease the malondialdehyde content. Compared with C0N0, under the treatments of C8N0, C16N0, C24N0, C0N150, C8N150, C16N150, C24N150, C0N300, C8N300, C16N300, and C24N300, the malondialdehyde content decrease by 1.11, 1.71, 5.84, 5.54, 7.23, 7.95, 8.72, 4.94, 5.81, 6.34 and 6.94 μmol g^−1^ in 2019, and 0.87, 3.59, 4.48, 5.25, 5.99, 8.41, 8.65, 2.59, 5.23, 6.12 and 6.82 μmol g^−1^ in 2020.

### Effects of bio-char and nitrogen fertilizer on superoxide dismutase activity of maize under different irrigation methods

**Figure 2:**
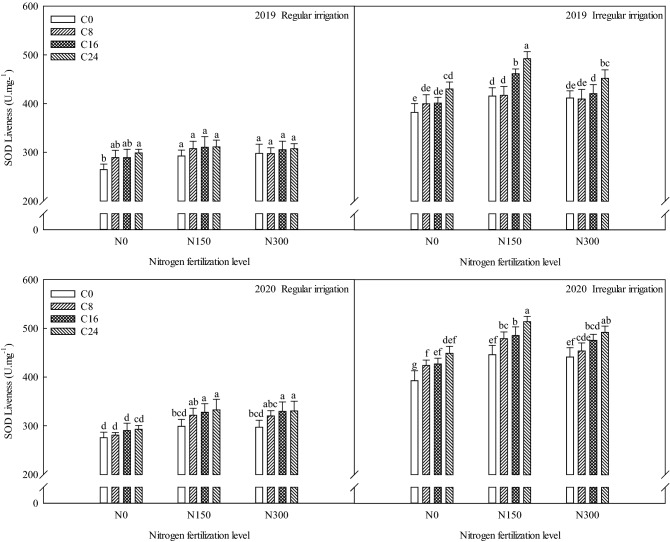
Effects of bio-char and nitrogen fertilizer on SOD activity of maize under different irrigation methods.

According to Fig. 2, under the regular irrigation condition, superoxide dismutase activity stayed in a stable range. Under the irregular irrigation condition, the nitrogen fertilization levels and bio-char levels significantly influenced (p ≤ 0.05) the superoxide dismutase activity compared to C0N0, resulting in the superoxide dismutase activity values that were higher than that of C0N0. Nitrogen and bio-char supply improve the superoxide dismutase activity. Compared with C0N0, under the treatments of C8N0, C16N0, C24N0, C0N150, C8N150, C16N150, C24N150, C0N300, C8N300, C16N300, and C24N300, the superoxide dismutase activity improved by in 0.27%, 5.24%, 12.86%, 8.92%, 9.44%, 20.99%, 29.13%, 7.87%, 7.34%, 10.23%, and 18.63% in 2019, and 8.16%, 8.92%, 14.53%,13.77%, 22.19%, 23.72%, 31.12%, 12.50%, 15.81%, 21.17%, and 25.51% in 2020.

### Effects of bio-char and nitrogen fertilizer on peroxidase activity of maize under different irrigation methods

**Figure 3:**
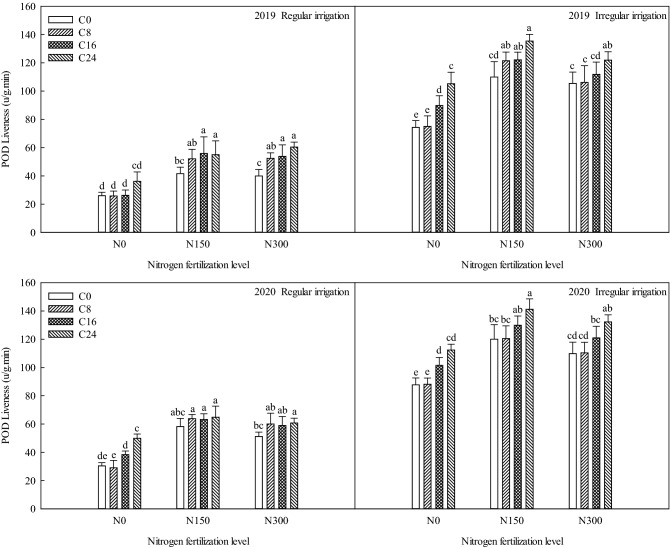
Effects of bio-char and nitrogen fertilizer on POD activity of maize under different irrigation methods.

As shown in Fig. 3, under the irregular irrigation condition, the nitrogen fertilization levels and bio-char levels significantly influenced (p ≤ 0.05) the peroxidase activity compared to C0N0, resulting in the peroxidase activity values that were higher than that of C0N0. Nitrogen and bio-char supply improve the peroxidase activity. Compared with C0N0, under the treatments of C8N0, C16N0, C24N0, C0N150, C8N150, C16N150, C24N150, C0N300, C8N300, C16N300, and C24N300, the peroxidase activity improved by 0.70, 15.49, 30.90, 35.70, 47.15, 47.78, 61.11, 31.11, 31.95, 37.57 and 47.57 μ g^−1^ min^−1^ in 2019, and 0.35, 13.79, 24.92, 32.33, 32.75, 42.12, 53.48, 22.02, 22.64, 33.27 and 44.52 μ g^−1^ min^−1^ in 2020.

### Effects of bio-char and nitrogen fertilizer on dry matter of maize under different irrigation methods

**Figure 4:**
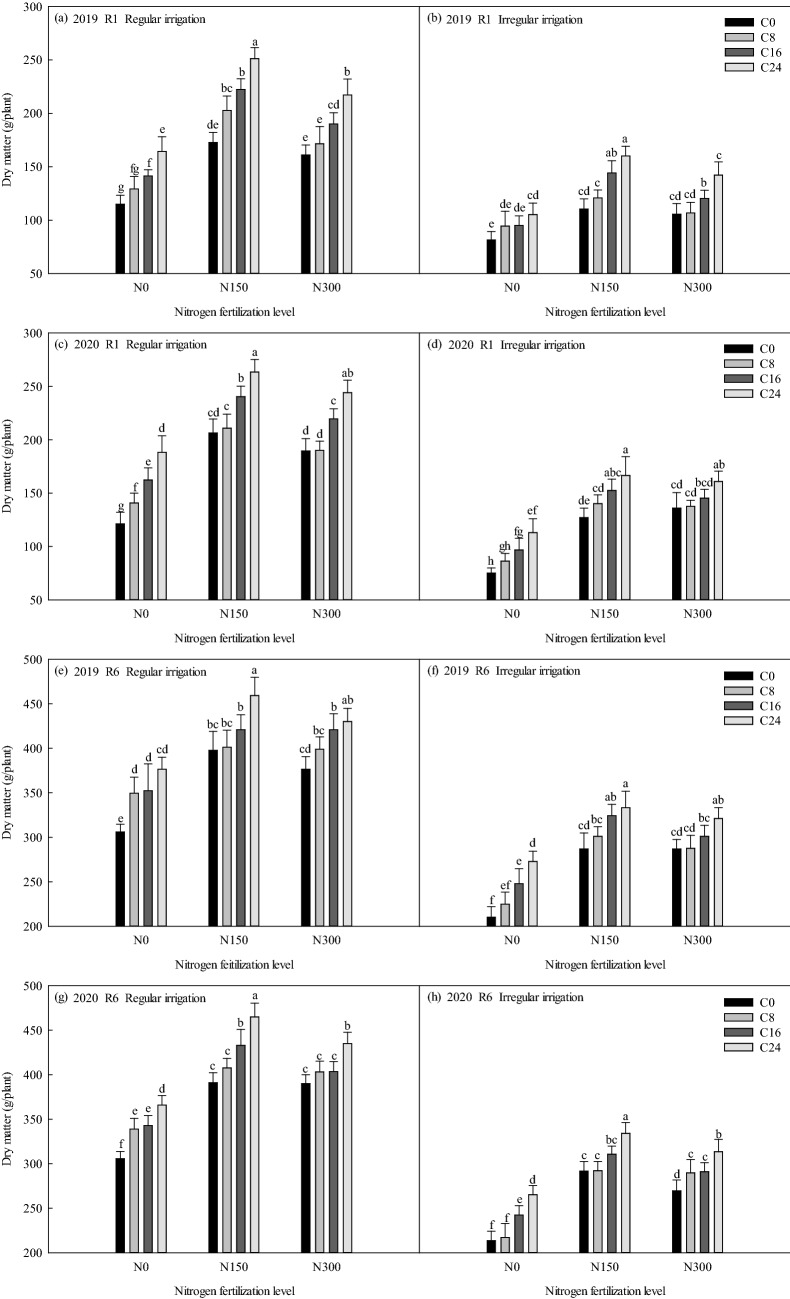
Effects of bio-char and nitrogen fertilizer on dry matter of maize under different irrigation methods.

It can be seen from Fig. 4, that the irrigation methods, nitrogen fertilization levels, and bio-char levels significantly influenced (p ≤ 0.05) the maize dry matter accumulation compared to C0N0, resulting in the dry matter values that were higher than that of C0N0. Nitrogen and bio-char supply increased dry matter accumulation. Compared with C0N0, under the treatments of C24N150, C24N300 and C24N0, the dry matter of the R1 stage increased by 136.24, 102.30, 49.50 g plant^−1^ in regular irrigation (Fig. 4a) and 78.57, 60.64, 23.66 g plant^−1^ in irregular irrigation (Fig. 4b) in 2019; the dry matter increased by 142.24, 122.99, 67.02 g plant^−1^ in regular irrigation (Fig. 4c) and 91.41, 85.78, 37.97 g plant^−1^ in irregular irrigation (Fig. 4d) in 2020. Compared with C0N0, under the treatments of C24N150, C24N300, and C24N0, the dry matter of the R6 stage increased by 153.30, 124.19, 70.47 g plant^−1^ in regular irrigation (Fig. 4e), and 123.01, 110.90, 62.65 g plant^−1^ in irregular irrigation (Fig. 4f) in 2019; the dry matter increased by 159.30, 129.38, 60.27 g plant^−1^ in regular irrigation (Fig. 4g) and 120.63, 99.98, 51.77 g plant^−1^ in irregular irrigation (Fig. 4h) in 2020.

In short, under the same irrigation method, the increase of maize dry matter from R1 to R6 improved significantly with the nitrogen level, nitrogen fertilizer could improve the maize dry matter accumulation ability. The maize dry matter of R1 to R6 increased significantly after the treatment of C24 compared to C0 under the same nitrogen and irrigation supply. The promotion effect of irrigation methods, nitrogen fertilizer, and bio-char on maize dry matter increased from year to year.

### Effects of bio-char and nitrogen fertilizer on maize yield under different irrigation methods

**Figure 5:**
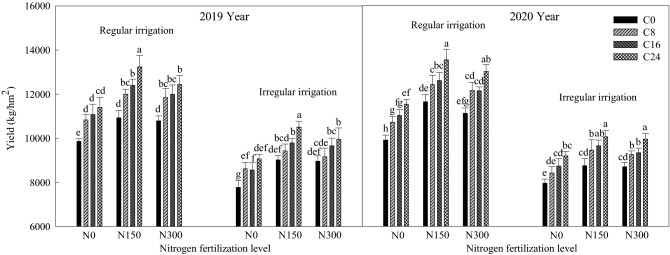
Effects of bio-char and nitrogen fertilizer on maize yield under different irrigation methods.

It can be seen from Fig. 5, that the irrigation methods, nitrogen fertilization levels, and bio-char levels significantly influenced (p ≤ 0.05) the maize yield compared to C0N0, resulting in maize yield values that were higher than those of C0N0. Nitrogen and bio-char supply increased maize yield. Compared to C0N0, under the treatments of C24N150, C24N300 and C24N0, maize yield in 2019 increased by 34.24%, 26.17%, 15.67% in regular irrigation and 35.13%, 27.99%, 16.60% in irregular irrigation; maize yield in 2020 increased by 36.45%, 31.16%, 16.33% in regular irrigation and 26.22%, 25.09%, 15.47% in irregular irrigation.

Under the same irrigation methods, the maize yield among treatments was as follows: N150 > N300 > N0, and under the same irrigation methods and nitrogen fertilization levels, the maize yield among treatments was as follows: C24 > C16 > C8 > C0. The treatment of C24N150 recorded the highest average yield in the 2-year test, which was 13,229.15 kg h^−1^ m^−2^ in 2019 and 13,541.70 kg h^−1^ m^−2^ in 2020. The maize yield in different planting years showed as follows: 2020 > 2019, which indicated that the promotion effect of nitrogen fertilizer and bio-char on maize yield increased from year to year.

### Correlation analysis of endogenous protective enzymes, dry matter accumulation and yield of maize

As shown in Table 3, superoxide dismutase activity was significantly correlated with peroxidase activity and malondialdehyde content, while negatively correlated with dry matter and yield. Peroxidase activity was significantly correlated with malondialdehyde content in 2019 and positively correlated with malondialdehyde content in 2020. Superoxide dismutase activity, peroxidase activity, and malondialdehyde content were negatively correlated with dry matter accumulation and yield, the results showed the decrease of superoxide dismutase activity, peroxidase activity, and malondialdehyde content could significantly improve maize dry matter. Dry matter was significantly correlated with maize yield, indicating that the increase in dry matter accumulation could significantly improve maize yield. The correlation coefficients of superoxide dismutase activity, peroxidase activity, malondialdehyde content, dry matter at the R1 stage, and dry matter at the R6 stage with yield were − 0.633, − 0.535, − 0.903, 0.957 and 0.979 in 2019, and − 0.654, − 0.597, − 0.888, 0.950 and 0.979 in 2020. The results showed that the correlation between the two experimental indexes was consistent, so the repeatability was precise.

**Table 3:** Correlation analysis of endogenous protective enzymes, dry matter accumulation and yield of maize.

Year	Index	SOD activity (U mg^−1^)	POD activity (μ g^−1^ min^−1^)	MDA Content (μmol g^−1^)	Dry matter in R1 (g plant^−1^)	Dry matter in R6 (g plant^−1^)	Yield (kg h^−1^ m^−2^)
2019	SOD activity (U mg^−1^)	1					
	POD activity (μ g^−1^ min^−1^)	0.968**	1				
	MDA Content (μmol g^−1^)	0.633**	0.534**	1			
	Dry matter in R1 (g plant^−1^)	− 0.489*	− 0.379 ns	− 0.813**	1		
	Dry matter in R6 (g plant^−1^)	− 0.613**	− 0.493*	− 0.919**	0.953**	1	
	Yield (kg h^−1^ m^−2^)	− 0.633**	− 0.535**	− 0.903**	0.957**	0.979**	1
2020	SOD activity (U mg^−1^)	1					
	POD activity (μ g^−1^ min^−1^)	0.987**	1				
	MDA Content (μmol g^−1^)	0.568**	0.513*	1			
	Dry matterin R1 (g plant^−1^)	− 0.461*	− 0.384 ns	− 0.853**	1		
	Dry matter in R6 (g plant^−1^)	− 0.591**	− 0.519**	− 0.914**	0.974**	1	
	Yield (kg h^−1^ m^−2^)	− 0.654**	− 0.597**	− 0.888**	0.950**	0.979**	1

## Discussion

The change of endogenous protective enzymes is an important reaction in the physiological activities of plant resistance to stress^35^. The study of endogenous protective enzymes is of great significance to elucidate how plants maintain normal growth and development under the water shortage condition ^36^. Higher yield and biomass can be obtained by maintaining higher superoxide dismutase activity, peroxidase activity, and lower malondialdehyde content ^13,37,38^. Zhang showed that the content of malondialdehyde in leaves increased significantly after drought stress ^39^, and the superoxide dismutase and peroxidase could reduce the content of malondialdehyde and remove reactive oxygen species, to protect the plasma membrane system from membrane lipid peroxidation ^40^. Studies have found that the activities of superoxide dismutase and peroxidase in leaves increased significantly with the application of bio-char^41,42^ and nitrogen fertilizer ^43^. In the research presented here, under the irregular irrigation condition, compared with C0N0, superoxide dismutase activity of C24N0, C24N150, and C24N300 treatments increased by 12.86–14.53%, 29.13–31.12%, 18.63–25.51%, respectively. The activities of endogenous protective enzymes increased after the treatment of N150 under the same irrigation method. The activities of endogenous protective enzymes increased significantly with the bio-char levels under the same nitrogen fertilization level.

Dry matter accumulation is the key to yield formation of maize ^44^. Studies have shown that bio-char promoted dry matter accumulation, and different bio-char levels had different effects on dry matter accumulation^45–47^. Nitrogen is one of the essential nutrients for maize growth, which plays an indispensable role in promoting the accumulation of dry matter and yield formation of maize ^48^. Li^49^ found that there was a significant positive correlation between maize dry matter and yield, and proper nitrogen is beneficial to dry matter accumulation and yield formation of maize^50,51^. At a certain level of nitrogen fertilizer application, dry matter accumulation and yield of maize improved with the increase of bio-char application. In this study, under the irregular irrigation condition, compared with C0N0, dry matter in the R1 stage of C24N0, C24N150, and C24N300 treatments increased by 23.66–37.97, 78.57–91.41, 60.64–85.78 g plant^−1^, respectively. Compared with C0N0, dry matter in the R6 stage of C24N0, C24N150, and C24N300 treatments increased by 51.77–62.65, 120.63–123.01, 99.08–110.90 g plant^−1^, respectively. The results showed that N150 promoted the dry matter accumulation of maize the most at R1 and R6 stages within nitrogen fertilization levels. The improving effect of dry matter improved significantly with the increase of bio-char levels.

Su ^52^ showed that bio-char can significantly increase maize yield, and bio-char application had a long-term effect on the increase of yield ^53,54^. A range of studies has found that nitrogen fertilizer can increase the yield^55,56^. In this paper, under the irregular irrigation condition, compare to C0N0, the yield of C24N0, C24N150, and C24N300 treatments increased by 15.47–16.00%, 26.22–35.13%, 25.09–27.99%, respectively. The yield of different treatments under the same irrigation condition was as follows: C24N150 > C24N300 > C16N150 > C16N300 > C8N150 > C8N300 > C24N0 > C0N150 > C0N300 > C8N0 > C16N0 > C0N0. The yield increased significantly after the treatment of N150 under the same irrigation method. The yield improved with the increase of bio-char levels (0–24 t h^−1^ m^−2^) under the same nitrogen fertilization level.

In conclusion, this experiment focused on the effects of bio-char and nitrogen fertilization levels on the endogenous protective enzyme, dry matter, and yield of maize under different irrigation methods in 2019–2020. The results showed that the response of endogenous protective enzymes, dry matter, and yield of maize to bio-char and nitrogen supply were significant. The promotion effect of bio-char and nitrogen fertilizer on the above indexes increased from year to year. The effect of regular irrigation was greater than irregular irrigation. Under the same irrigation methods, the endogenous protective enzymes, dry matter accumulation, and yield of maize showed the best performance under the treatment of C24N150. The values of the above indexes were positively correlated with irrigation, nitrogen, and bio-char supply. Considering the reality of the drought in Inner Mongolia during maize cultivation, the treatment of C24N150 reached the maximum yield of maize planting. The maximum yield of maize was 10,048.65 kg h^−1^ m^−2^, which was 2094.11 kg h^−1^ m^−2^ more than that of C0N0.

## Conclusion

On average, the superoxide dismutase activity, peroxidase activity, dry matter accumulation, and yield of maize planting were significantly increased by nitrogen fertilizer and bio-char supply, while the malondialdehyde content decreased by nitrogen fertilizer and bio-char supply. The promotion effect of nitrogen fertilizer and bio-char on the above indexes increased from year to year. Conclusion in this study, C24N150 was selected as the most effective treatment to improve the endogenous protective enzyme activities, dry matter accumulation, yield, and decrease malondialdehyde content of maize, whether in regular irrigation conditions or irregular irrigation conditions. This means that the treatment of C24N150 could promote the growth and yield formation of maize under regular irrigation conditions, however, under the condition of irregular irrigation seriously affecting maize growth and development, the treatment of C24N150 still could enhance the endogenous protective enzyme activities, improved the drought resistance of maize, and then maximize the dry matter accumulation and yield of maize.
